# Integrated Phosphoproteomics Identifies TGFβ-Dependent Phosphorylation Events Linking Kinase Signaling to Autophagy in Palatogenesis

**DOI:** 10.3390/proteomes14010005

**Published:** 2026-01-23

**Authors:** Xia Peng, Jing Chen, Xiaoyu Zheng, Xige Zhao, Yijia Wang, Xiaotong Wang, Juan Du

**Affiliations:** 1 Laboratory of Orofacial Development, Laboratory of Molecular Signaling and Stem Cells Therapy, Beijing Key Laboratory of Tooth Regeneration and Function Reconstruction, Beijing Stomatological Hospital, Capital Medical University, No. 9 Fanjiacun Road, FengTai District, Beijing 100070, China; 122020010406@ccmu.edu.cn (X.P.); chenjingecho629@163.com (J.C.);; 2 Department of Stomatology, Beijing Anzhen Hospital, Capital Medical University, No. 2 Anzhen Road, Chaoyang District, Beijing 100029, China; 3 Department of Geriatric Dentistry, Beijing Stomatological Hospital, Capital Medical University, No. 9 Fanjiacun Road, FengTai District, Beijing 100070, China

**Keywords:** mouse embryonic palatal mesenchyme (MEPM) cell, TGFβ2, phosphoproteomics, autophagy, palatogenesis

## Abstract

Background: Cleft palate (CP) is a prevalent craniofacial malformation, with the TGFβ pathway playing a critical role. Recent evidence links autophagy to regulating mouse embryonic palatal mesenchyme (MEPM) cells, but its interaction with TGFβ-activated phosphorylation cascades remains largely unknown. Here, we investigated the interplay between these pathways during palatogenesis. Methods: H&E and IHC analyses revealed increased expression of Beclin 1 and LC3 during the critical period of palatal shelf elevation and fusion (E13.5–E15.5). Bulk RNA sequencing (Bulk RNA-seq) further revealed enrichment of autophagy-related pathways and their interaction with TGFβ signaling. TMT-based phosphoproteomics was performed on TGFβ2-treated MEPM cells. Results: We identified 23,471 peptides and 3952 proteins, including 6339 phosphopeptides corresponding to 2195 phosphoproteins. Differential analysis found 477 phosphopeptides with increased abundance and 53 with decreased abundance, revealing the enrichment of seven serine (p-Ser) motifs (RxxS, SDxD, SDxE, SP, SxDE, SxEE, SxxxxD) and one threonine (p-Thr) motif (TP). Notably, kinase-substrate enrichment analysis identified CSNK2A as a previously unrecognized phosphorylation regulator, together with MAPKs and CDKs. Functional enrichment showed significant involvement of mTOR, MAPK, and autophagy/mitophagy pathways. Conclusions: Our findings revealed that TGFβ2 reshapes the MEPM phosphoproteome through Smad-independent pathway, expanding the palate-specific phospho-signaling atlas beyond the canonical Smad cascade.

## 1. Introduction

Cleft palate (CP) is one of the most common congenital malformations, occurring at 0.33 per 1000 deliveries [1]. It imposes significant physiological and economic burdens on affected individuals and their families [2]. The pathogenesis of CP is complex. Current research indicated that it is a multifactorial trait arising from gene–environment crosstalk [3,4]. Genetically, transforming growth factor-beta (TGFβ), Wnt, Notch and Hedgehog (Hh) modules are the most enriched sets in CP [5]. Environmentally, in addition to classic factors like smoking, alcohol consumption, and drugs, *P. gingivalis* and energy imbalances have also been confirmed to cause CP [6,7]. These findings underscore the multifactorial nature of CP pathogenesis. Owing to the similarity of palatal development between mice and humans, murine models are widely used to study CP [8]. Palatal development in mice begins at embryonic day 12.5 (E12.5) and ends at E18.5 [9]. During this period, the palatal shelves undergo rapid growth, elevation, fusion, and ossification [10]. Failure of any step in these signaling cascades arrests shelf fusion, making it essential to investigate the mechanism involved in palatogenesis.

Autophagy is an evolutionarily conserved process and a crucial mechanism for eukaryotes to maintain homeostasis [11]. Starvation, nutrient deficit and rapamycin serve as potent inducers of autophagic flux in multiple cell types [12]. Under the action of Beclin 1 and ATG, autophagosomes form [13]. Subsequently, lysosome-associated membrane protein 2 (LAMP2) licenses membrane fusion between autophagic vacuoles and lysosomes, thereby converting them into single-membrane autolysosomes [14]. Finally, under the action of microtubule-associated protein 1 light chain 3 alpha (LC3), the trapped proteins or organelles are broken down and reused inside the autolysosome [15]. During early mammalian gestation, autophagy delivers nutrients to the embryo and facilitates implantation and pregnancy continuation [16,17]. Our previous research showed that inhibiting autophagy with chloroquine (CQ) suppressed the proliferation and promoted the apoptosis of mouse embryonic palatal mesenchymal (MEPM) cells [18]. In addition, our study also revealed that ROS/ERK pathway-mediated autophagy could protect against nicotine-induced apoptosis in MEPM cells [19]. However, the activation process and specific regulatory mechanism of autophagy in MEPM cells remain unclear.

TGFβ signaling is crucial for normal craniofacial morphogenesis. Perturbation of TGFβ signaling may result in various malformations, including CP. The TGFβ pathway coordinates multiple events: growth, proliferation, apoptosis, epithelial-to-mesenchymal transition (EMT), and autophagy [15,20]. Emerging studies links autophagy to palate formation [18,19,21]. In canonical TGFβ signaling, the TβRII/TβRI complex phosphorylates R-SMADs; together with SMAD4 they enter the nucleus and boost autophagy-related genes [22]. Even if R-SMADs act alone, SMAD4 knockdown blocks TGFβ-driven autophagy [23]. Remarkably, the recent report revealed functional overlap between ectodermal SMAD4 and p38, shifting focus to SMAD-independent branches as key autophagy regulators [24]. Above studies illustrated that TGFβ can affect autophagy via SMAD and SMAD-independent pathways [22,23,24]. However, the specific mechanism by which TGFβ induces autophagy in MEPM cell is still unclear.

Phosphorylation cascades play a pivotal role in intracellular signal transduction, acting as molecular switches that amplify and integrate extracellular cues [25]. Through sequential phosphorylation events, cells can rapidly modulate protein activity, stability, and localization, thereby ensuring precise regulation of developmental or pathological processes. Within this context, the TGFβ signaling pathway exerts its effects largely through phosphorylation-dependent mechanisms, particularly the activation of receptor-regulated SMADs, which subsequently drive downstream transcriptional programs [26]. In addition, the effect of TGFβ signaling on protein phosphorylation during palate development is still unclear.

Based on this, we used phosphoproteomics to study the changes in phosphorylation modifications in E13.5 MEPM cells under the action of TGFβ2. Through this study, we hope to systematically reveal the regulatory features of protein phosphorylation modifications by TGFβ2 signaling during palate development, clarify the potential molecular link between TGFβ and autophagy, thereby providing a conceptual framework for future risk assessment, mechanistic classification, or early biomarker discovery in developmental disorders.

## 2. Materials and Methods

### 2.1. Animals

C57BL/6 mice were obtained from SPF (Beijing) Biotechnology Co., Ltd. (Beijing, China). The mice were maintained in a specific pathogen-free (SPF) facility under controlled environmental conditions, maintained at a temperature of 22 ± 2 °C and a relative humidity of 55 ± 5%, with a 12 h light/dark cycle. Standard laboratory chow and water were supplied ad libitum. After one week of acclimatization, healthy female C57BL/6 mice (8–10 weeks old; 22–25 g) were mated overnight with healthy male mice (10–12 weeks old) at a ratio of 2:1. Vaginal plug was checked the following morning, and that day was designated as embryonic day 0.5 (E0.5). Pregnancy was confirmed at E13.5, E14.5, and E15.5 by observing a weight gain of more than 4 g compared to E0.5.

Pregnant mice were euthanized by cervical dislocation, and fetal heads were harvested following our previously reported protocols [18]. This study strictly adhered to the ARRIVE guidelines, and all efforts were made to minimize animal suffering. All animal experiments were conducted in strict compliance with the Guidelines for the Care and Use of Laboratory Animals and were approved by the Laboratory Animal Management Committee of Beijing Stomatological Hospital, Capital Medical University (Approval No. KQYY-2022-08-003, approved on 2 August 2022). All experimental personnel were qualified for animal experimentation (Animal Use Permit No. 1123042400023).

### 2.2. Isolation and Culture of E13.5 MEPM Cells

Pregnant mice at E13.5 were obtained as described above. The fetal heads were separated using ophthalmic scissors along the line connecting the tragus and the labial commissure. The palatal shelves were dissected horizontally, immediately placed in ice-cold sterile phosphate-buffered saline (PBS), and transferred to a sterile laminar flow hood.

The tissues were digested with 0.25% Trypsin-EDTA (Cat. No. 25200-072, Gibco, Grand Island, NY, USA) at 37 °C for 30 min. The digested cells (MEPM cells) were cultured in DMEM/F12 medium (Cat. No. SH30022.01, HyClone, Logan, UT, USA) supplemented with 10% fetal bovine serum (Cat. No. 10099-141, Gibco, Grand Island, NY, USA) and 1% penicillin/streptomycin at 37 °C in a humidified atmosphere containing 5% CO_2_. Cells were subcultured when they reached approximately 80–90% confluence. Cells at passage 2 (P2) were used for all subsequent experiments to ensure consistency. For the treatment groups, Recombinant Mouse TGF-beta 2 Protein (Cat. No. 7346-B2-005, R&D Systems, Minneapolis, MN, USA) was reconstituted according to the manufacturer’s instructions. MEPM cells were treated with 10 ng/mL TGFβ2 for 0, 6, 12, or 24 h prior to protein extraction for Western blot or proteomic analysis.

### 2.3. Hematoxylin and Eosin (H&E) Staining

Following the procedures outlined in our previous study [27], fetal mouse heads were collected at E13.5, E14.5, and E15.5 (*n* = 3 per group). The tissues were fixed in 4% paraformaldehyde (PFA) and dehydrated. Subsequently, the samples were embedded in paraffin and sectioned at 5 μm thickness. The sections were deparaffinized and rehydrated using graded ethanol series. Sections were stained with hematoxylin for 3 min, rinsed, and differentiated with 1% hydrochloric acid-alcohol. After counterstaining with eosin for 1 min, the slides were then dehydrated through graded ethanol, cleared in xylene, and mounted with neutral balsam. Images were acquired using an automated pathological slide scanner, and regions of interest were selected using an image viewer (Shengqiang Technology, Shenzhen, China).

### 2.4. Immunohistochemistry (IHC) Staining

Paraffin-embedded sections (5 μm thickness) were prepared, deparaffinized in xylene, and rehydrated through a graded ethanol series. For antigen retrieval, sections were incubated in sodium citrate buffer (Cat. No. BL619A, Biosharp, Beijing, China) at 95 °C for 25 min. After cooling to room temperature, endogenous peroxidase activity was blocked using Endogenous Peroxidase Blocking Solution (Cat. No. ZLI-9311D, ZSGB-BIO, Beijing, China) for 15 min at room temperature. Non-specific binding was blocked with 10% goat serum for 60 min at room temperature. The sections were then incubated with primary antibodies against Beclin 1 (1:2000; Cat. No. 3494, Cell Signaling Technology) and LC3 (1:5000; Cat. No. 2775, Cell Signaling Technology) overnight at 4 °C. Following standard washing steps, the sections were incubated with the secondary antibody detection system (Cat. No. SAP-9100, ZSGB-BIO, Beijing, China) according to the manufacturer’s instructions. Immunoreactivity was visualized using a DAB detection kit (Cat. No. PV-6000D, ZSGB-BIO, Beijing, China). The sections were counterstained with hematoxylin, dehydrated, cleared in xylene, and mounted with neutral balsam. Images were acquired using an automated pathological slide scanner, and regions of interest were selected using an image viewer (Shengqiang Technology, Shenzhen, China). IHC Analyses were performed in a blinded manner.

### 2.5. Western Blot

MEPM cells were cultured in the medium containing 10 ng/mL TGFβ2 for 0, 6, 12, and 24 h (*n* = 3 per group). Total protein extraction, quantitative methods, and SDS polyacrylamide gel tests were performed according to a previous protocol [28]. Membranes were blocked with 5% non-fat milk or 5% BSA in TBST for 1 h at room temperature and then incubated with primary antibodies overnight at 4 °C. After washing, the membranes were incubated with HRP-conjugated secondary antibodies for 1 h at room temperature. All antibodies were listed in Table 1.

### 2.6. Bulk RNA Sequencing (Bulk RNA-Seq) Analysis

The transcriptomic data we used was obtained from the NCBI database (accession number: PRJNA1027758), featuring palatal shelves from E13.5, E14.5, and E15.5 mouse embryos (*n* = 6 per group). In the Bulk RNA-seq, we set a fold change ≥2 (i.e., the absolute value of log2FC ≥ 1) and a q-value < 0.05 (the q-value is the corrected value of the *p*-value) as the threshold criteria for screening differentially expressed genes (|log2FC| ≥ 1 and q < 0.05), and then performed enrichment analysis on the obtained data. Mfuzz analysis was performed using the OmicStudio tools (LC-Bio, Hangzhou, China) at https://www.omicstudio.cn/tool (last accessed on 15 May 2024). Clusterprofiler KEGG enrichment Cnet graph was plotted by https://www.bioinformatics.com.cn (last accessed on 10 December 2024), an online platform for data analysis and visualization. The protein complex network was created using Metascape (https://metascape.org/, accessed on 1 July 2025) [29].

### 2.7. Tandem Mass Tag (TMT) Labeling and Phosphopeptides Enrichment

For our quantitative phosphoproteomic analysis, we used 6 samples divided into a control group (*n* = 3 biological replicates) and a TGFβ2-treated group (*n* = 3 biological replicates). Peptide mixture (100 μg) was labeled using TMT reagent according to the manufacturer’s instructions.

Phosphopeptides were enriched using a two-step strategy [30]. Briefly, the TMT-labeled peptide mixture was first subjected to enrichment using the HiSelect TiO_2_ Phosphopeptide Enrichment Kit (A32993, Thermo Fisher Scientific, Waltham, MA, USA) to preferentially capture multiphosphorylated peptides. Peptides were loaded and washed with an aqueous solution containing 2% acetonitrile and 0.1% trifluoroacetic acid (TFA), and bound phosphopeptides were eluted with 50% acetonitrile containing 5% ammonia, following the manufacturer’s protocol.

The TiO_2_ flow-through and wash section were subsequently combined and subjected to a second enrichment using the HiSelect Fe-NTA Phosphopeptide Enrichment Kit (A32992, Thermo Fisher Scientific, Waltham, MA, USA) to enrich predominantly monophosphorylated peptides. The loading and washing conditions were identical to those used for TiO_2_ enrichment, and phosphopeptides were eluted with 0.5 M imidazole in 0.1% TFA. Non-specifically bound peptides were removed during the washing steps. Finally, eluates from both TiO_2_ and Fe-NTA enrichments were combined, dried by vacuum centrifugation at 45 °C, and resuspended in 0.1% formic acid for Liquid chromatography-tandem mass spectrometry (LC-MS/MS) analysis.

### 2.8. LC-MS/MS Analysis

The peptide mixtures were separated using an Easy-nLC system (Thermo Fisher Scientific, Waltham, MA, USA) coupled to a Q Exactive HF-X mass spectrometer (Thermo Fisher Scientific, Waltham, MA, USA). Peptides were loaded onto an Acclaim PepMap RSLC C18 column (50 µm × 15 cm, nanoViper, Thermo Fisher Scientific, Waltham, MA, USA) and separated with a 240 min gradient at a flow rate of 300 nL/min. The gradient comprised: 0–5 min, 5% solvent B (80% acetonitrile, 0.1% formic acid); 5–210 min, 5–28% B; 210–220 min, 28–38% B; 220–230 min, 38–99% B; and 230–240 min, 99% B.

The mass spectrometer functioned in positive ion mode using a data-dependent acquisition (DDA) strategy. Full MS scans were recorded in the Orbitrap mass analyzer over a range of 350–1800 *m*/*z* with a resolution of 120,000 (at *m*/*z* 200). The automatic gain control (AGC) target stood at 3 × 10^6^, and the maximum injection time (IT) was 50 ms. The top 10 most abundant precursor ions were selected for HCD fragmentation with a normalized collision energy (NCE) of 30 eV. MS2 spectra were acquired with a resolution of 45,000, an AGC target of 2 × 10^5^, a maximum IT of 120 ms, and an isolation window of 2.0 *m*/*z*. The dynamic exclusion duration was set to 30 s.

### 2.9. Gene Ontology (GO) Enrichment Analysis and Kyoto Encyclopedia of Genes and Genomes (KEGG) Pathway Enrichment Analysis

A list of all protein sequences was aligned to the NCBI database, and the GO term of the sequence with the highest Bit-Score was chosen. Then, protein annotation based on GO terms was initially performed using Blast2GO Command Line (Version 1.3.3, BioBam Bioinformatics, Valencia, Spain), followed by refinement and establishment of relationships among GO terms with ANNEX. All target genes were annotated with GO terms using the GO databases (http://www.geneontology.org/, accessed on 3 February 2023). Enrichment analysis of pathways was conducted with KEGG, and the resulting pathways were classified into hierarchical groups based on KEGG guidelines (http://www.genome.jp/kegg/, accessed on 3 February 2023). The bar and bubble charts from KEGG and GO pathway enrichment are presented by the OmicStudio platform (LC-Bio, Hangzhou, China; https://www.omicstudio.cn/tool, last accessed on 15 May 2024).

### 2.10. Gene Set Enrichment Analysis (GSEA) and Kinase-Substrate Enrichment Analysis (KSEA)

The significance of the pathway-level difference was computed using GSEA. For phosphoproteomic data, GSEA was conducted using the data set “mh.all.v2022.1.Mm.symbols.gmt [mouse-ortholog hallmark]” from the MSigDB [31]. The results of GSEA were visualized using GSEA v4.3.3 (Broad Institute, Cambridge, MA, USA; http://www.gsea-msigdb.org/gsea/downloads.jsp, accessed on 6 February 2024).

KSEA analysis was performed using different phosphopeptides and their phosphorylation modification sites using Signaling Network Open Resource (SIGNOR) database (https://signor.uniroma2.it/, accessed on 28 July 2021) and PhosphoSitePlus database to predict potential regulatory kinases upstream. KSEA algorithms were used to calculate the kinase activity on the KSEA website (Case Western Reserve University, Cleveland, OH, USA; https://casecpb.shinyapps.io/ksea/, accessed on 26 June 2017) and scored with a Z-score value. Z-score > 0 indicated that kinase activity was increased abundance. Z-score < 0 indicates decreased abundance. The higher the fraction, the higher the kinase activity.

Furthermore, phosphorylation motif analysis was conducted using the MoMo tool (Modification Motifs, MEME Suite software version 5.5.0, University of Nevada, Reno, NV, USA) to identify conserved consensus sequences. Pre-aligned peptide sets were extracted centered on the phosphorylation sites with ±6 amino acids flanking the modified residue.

### 2.11. Construction of Protein–Protein Interaction (PPI) Network

The PPI network of differentially abundant proteins (DAPs) was built on the STRING database v11.5 (https://string-db.org/, accessed on 19 February 2023) with a minimum required interaction score set to the medium confidence level (0.400). The network was visualized using Cytoscape v3.7.2 (https://cytoscape.org, accessed on 19 February 2023). We used Molecular Complex Detection (MCODE) to select hub modules, and cytoHubba to calculate the top 15 hub genes of PPI networks using eight different ranking algorithms (MNC, Degree, BottleNeck, EcCentricity, Closeness, Radiality, Betweenness, and Stress).

### 2.12. Statistical Analysis

For Proteomics Analysis: The raw data files (.raw) generated by the Q Exactive HF-X were processed using Proteome Discoverer 2.2 (Thermo Fisher Scientific, Waltham, MA, USA) and searched against the database using the MASCOT 2.6 server (Matrix Science, London, UK). Related parameters and descriptions are shown in Table 2. The data were filtered with a False Discovery Rate (FDR) < 0.01 at both peptide and protein levels to ensure high reliability. Phosphorylation site localization probabilities were calculated, and only sites with a probability > 0.75 were considered confident. Differentially abundant phosphopeptides (DAPPs) were identified based on the criteria of a fold change > 1.2 or <0.83 (corresponding to 1/1.2) and a *p*-value < 0.05 (Student’s *t*-test). Functional enrichment analysis (GO and KEGG) was performed using Fisher’s exact test (two-tailed), with a *p*-value < 0.05 considered statistically significant.

Statistical analyses for histological and Western blot data were performed using GraphPad Prism 10.4.1 (Accessed on 5 December 2024) (GraphPad Software, La Jolla, CA, USA). All quantitative data are presented as mean ± standard deviation (SD) from at least three independent biological replicates. Differences between two groups were analyzed using an unpaired Student’s *t*-test. For comparisons among three or more groups, a one-way analysis of variance (ANOVA) was performed, followed by Tukey’s post hoc test for multiple comparisons. A *p*-value < 0.05 was considered statistically significant.

## 3. Results

### 3.1. Autophagy Is a Key Event in Palate Development

**Figure 1:**
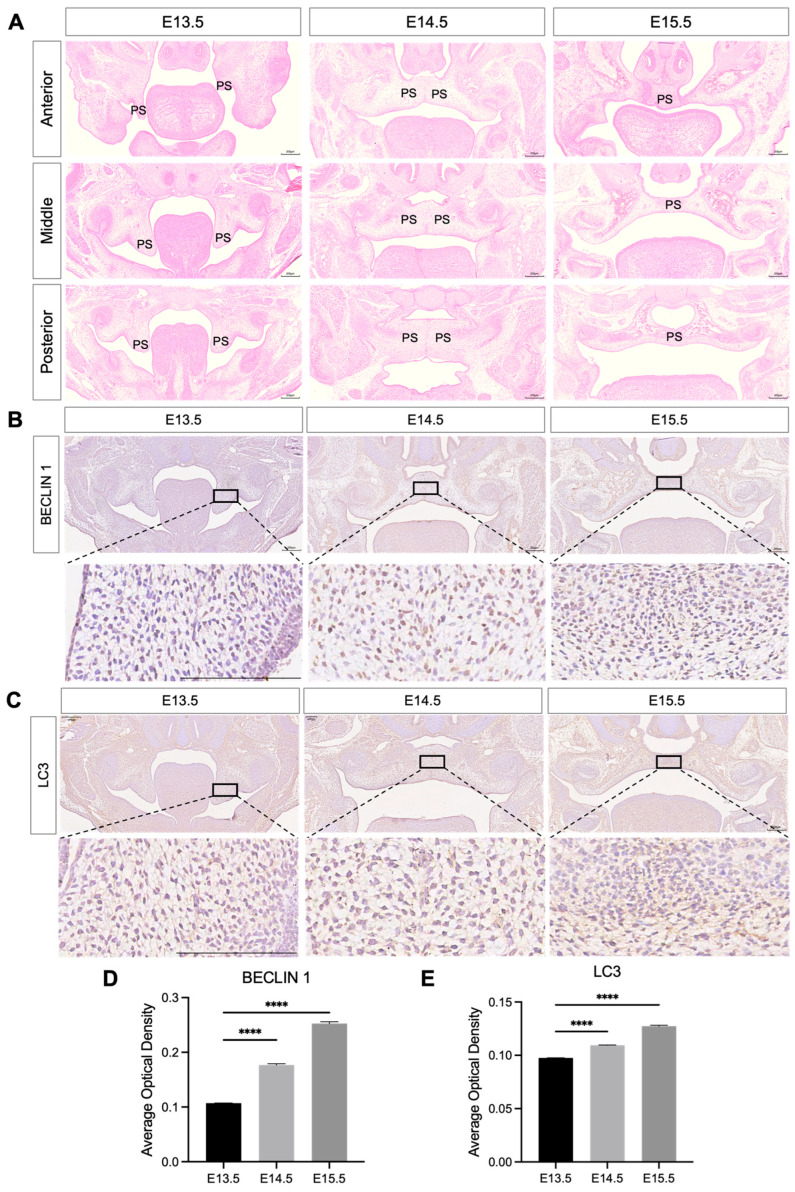
Morphological changes and autophagy protein level in the murine palatal shelves during development. (**A**) H&E staining of mouse palatal shelves from E13.5 to E15.5, showing developmental changes. PS, palatal shelves. Scale bar: 200 μm. (**B**,**C**) Immunohistochemical staining for autophagy markers BECLIN 1 and LC3 in palatal shelves at E13.5, E14.5, and E15.5. Brownish-yellow cytoplasmic staining indicates positive cells. Scale bar: 200 μm. (**D**,**E**) Quantification of the mean optical density from the immunohistochemistry, showing expression levels of BECLIN 1 (**D**) and LC3 (**E**). The data shown here are from representative experiments with 3 biological replicates and 3 technical replicates. Mean ± SD, **** *p* < 0.0001.

The formation of the fetal mouse palate shelves begins at E12.5, followed by vertical growth, elevation, contact, fusion, and ossification, with complete palate development by E18.5 [10]. Failure of palate elevation, contact, or fusion can lead to CP. The E13.5 to E15.5 period corresponds to this critical stage, dissecting its molecular events clarifies the precise spatiotemporal mechanisms of palatogenesis [32]. The H&E staining results showed that shelves were vertical at E13.5, fused by E14.5, and fully merged at E15.5, matching published timelines [9] (Figure 1A).

Autophagy plays a crucial role in early embryonic development, and its imbalance can lead to pregnancy complications such as pregnancy failure and fetal developmental disorders [17]. Beclin-1 is involved in the early stages of autophagy, and LC3 plays a role in autophagosome formation which are markers of autophagy [33]. We further investigated their expression patterns in palatogenesis. IHC staining results indicated that the expression levels of Beclin-1 and LC3 in the palatal mesenchyme were gradually upregulated from E13.5 to E15.5 in the palatal shelves (Figure 1B–E).

**Figure 2:**
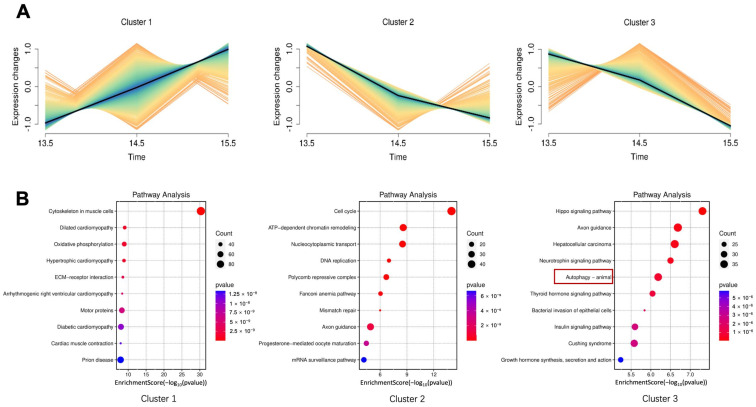
Mfuzz clustering identifies an autophagy regulated gene cluster. (**A**) Mfuzz soft clustering analysis was performed on DEGs identified from bulk RNA-seq of mouse palatal shelves at E13.5, E14.5, and E15.5. Genes were partitioned into 3 distinct clusters. The color gradient represents the membership score, with blue/cyan lines indicating high membership values and yellow/orange lines indicating low membership values. (**B**) Bubble plots showing the top enriched KEGG pathways for each cluster. Bubble size is proportional to the gene count, and color indicates the *p*-value.

To define how autophagy shapes palate morphogenesis, we utilized publicly available Bulk RNA-seq data (Accession number: PRJNA1027758) from E13.5 to E15.5 stages. First, we used the Mfuzz package to classify differentially expressed genes (DEGs) from different days into three patterns (Figure 2A). Then, KEGG pathway enrichment was then applied to the genes in three modules. The bubble plot showed that genes in cluster 3 were enriched in autophagy (Figure 2B).

**Figure 3:**
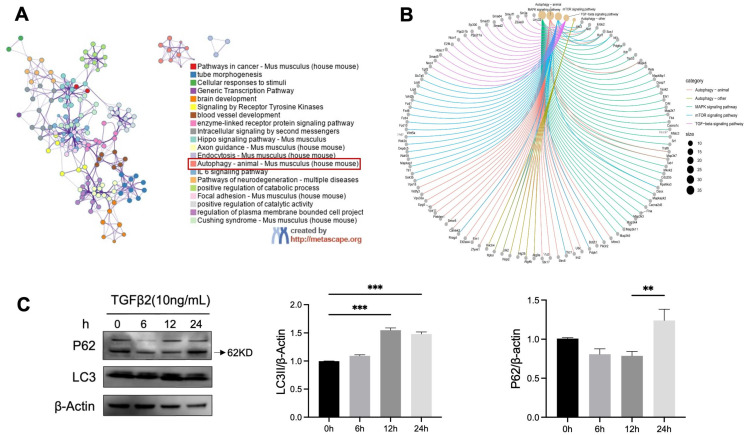
Bioinformatic analysis reveals a pro-autophagic gene cluster regulated by TGFβ2 during palatogenesis. (**A**) An enrichment network of genes from Cluster 3 was constructed using Metascape. Nodes are colored by their membership in enriched functional clusters, representing distinct biological pathways. (**B**) Cnet plot visualizing the relationships between the most significant KEGG pathways and their corresponding genes within Cluster 3. (**C**) To validate the role of TGFβ signaling in autophagy, MEPM cells were treated with TGFβ2. Western blot analysis showed the protein levels of LC3 and P62 in MEPM cells treated with 10 ng/mL TGFβ2 for 0, 6, 12, and 24 h. P62 was detected as two closely migrating bands, which likely represent different post-translationally modified forms. Quantification was performed using the lower ~62 kDa P62 band, which represents the canonical form. Data are expressed from 3 biological replicates (**A**,**B**) or 3 biological replicates with 3 technical replicates each (**C**). Mean ± SD, ** *p* < 0.01, and *** *p* < 0.001.

We then used Metascape to observe the gene-pathway correlation of genes in cluster 3 and found that the autophagy pathway was also enriched (Figure 3A). We then used a Cnet network diagram to display the significantly altered pathways and related genes in cluster 3. We found that in addition to autophagy and its related mTOR and MAPK pathways, genes related to the TGFβ pathway were also enriched in this module (Figure 3B). The synergistic action of these two pathways led us to speculate on a potential connection between them. Thus, we further investigated the relationship between the TGFβ signaling pathway and autophagy.

### 3.2. TGFβ2 Induced Autophagy in MEPM Cells

Based on our transcriptomic findings, we hypothesized that TGFβ signaling could directly induce autophagy. To validate this in vitro, we treated MEPM cells with TGFβ2 and investigated standard autophagy markers, including the LC3 II/I ratio and the expression of P62 [33]. Our study demonstrated that TGFβ2 treatment led to autophagy in a time-dependent manner (Figure 3C), as shown by significant increases in the LC3 II/I ratio, alongside the suppression of P62. The significant enhancement of autophagy observed at 12 h with 10 ng/mL of TGFβ2 justified the use of this condition for our subsequent mechanistic analysis.

To understand the molecular mechanism behind this phenomenon, we focused on phosphorylation. The TGFβ signaling pathway, a critical regulator of palate development [34], depends on phosphorylation to activate downstream signals through both canonical Smad and non-canonical pathways [35,36]. Therefore, to identify the key signaling events linking TGFβ2 to the induction of autophagy, we proceeded to examine the changes in the phosphoproteome of MEPM cells upon TGFβ2 activation.

### 3.3. Phosphorylation Characteristics and Identification of Differentially Phosphorylated Peptides/Proteins in MEPM Cells Under TGFβ2 Activation

Quantitative phosphoproteomics is often used to detect the phosphorylation status of proteins within cells under external stimuli. This is an integrated technology that combines LC-MS/MS with TMT labeling [37]. In our experiment, we used TMT reagents 126–131 to label MEPM cells in the control and TGFβ2-treated groups, respectively. After enrichment of phosphopeptides with TiO_2_ and high-resolution mass spectrometry analysis, a quantitative study of differentially modified phosphopeptides was performed (Supplementary Figure S1A). Our SDS separation gels showed clear and rich bands without degradation (Supplementary Figure S1B). The peptide quality control data showed that the mass error was within 8 ppm (Supplementary Figure S1C), and the number of amino acid residues in most peptides ranged from 6 to 20 (Supplementary Figure S1D), indicating that the accuracy of the MS data met the experimental requirements.

**Figure 4:**
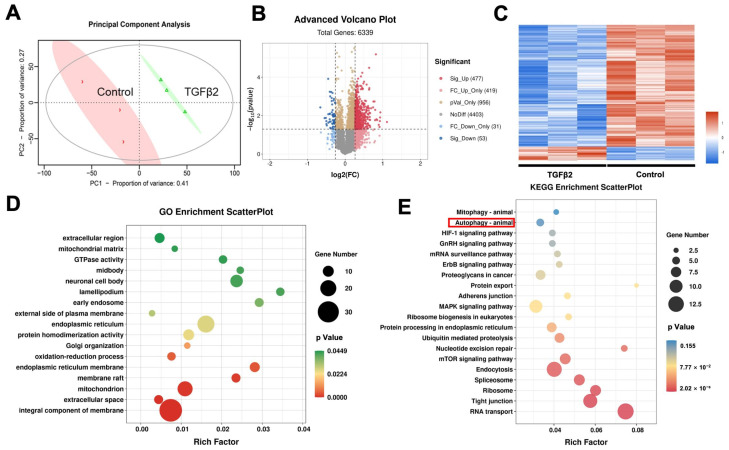
TGFβ2 treatment remodels the phosphoproteome of MEPM cells and activates autophagy-related signaling. Quantitative phosphoproteomic analysis was performed on MEPM cells treated with 10 ng/mL TGFβ2 for 12 h vs. control cells. (**A**) PCA plot showing distinct clustering between the control (red) and TGFβ2-treated groups (green). (**B**) Volcano plot illustrating significantly increased (red) and decreased (blue) abundance phosphoproteins. Dotted lines represent the statistical significance threshold. (**C**) Hierarchical clustering heatmap of differentially phosphorylated proteins, with red and blue indicating high and low phosphorylation levels, respectively. (**D**,**E**) GO and KEGG pathway enrichment analyses of the differentially phosphorylated proteins, displaying the most significant biological processes and pathways.

Principal component analysis (PCA) can intuitively show the overall clustering trend and inter-group differences among different samples [38]. Our results showed that there was a large inter-group difference between the control and TGFβ2-activated MEPM cells. The two groups showed different dispersion trends, and the intra-group dispersion was within the acceptable range (Figure 4A).

The LC-MS/MS analyses showed phosphorylation changes of phosphopeptides in the MEPM cells. A total of 3952 proteins and 23,471 peptides were identified, including 2195 phosphorylated proteins and 6339 phosphorylated peptides (Table 3). Furthermore, we investigated the number of phosphorylation sites on different proteins and peptides. Among the identified peptides, 81.7% carried one phosphosite, 14.9% two, and 3.0% three or more (Supplementary Figure S1E). We then evaluated the phosphorylation levels in each group. Furthermore, the results showed that threonine, serine, and tyrosine are the main amino acid residues where phosphorylation occurs (Supplementary Figure S1F). In addition, most proteins had less than five phosphorylation sites (Supplementary Figure S1G). Among them, serine accounts for more than 90% of these phosphorylated residues, while tyrosine accounts for only approximately 0.5%. Furthermore, we used a volcano plot and a heatmap to further show the differential distribution of phosphorylated proteins in TGFβ2-activated MEPM cells. Our results showed that 530 DAPPs (477 with increased abundance and 53 with decreased abundance) were identified between the TGFβ2 and the control groups (Figure 4B,C). Among these, 351 phosphoproteins (314 with increased abundance and 37 with decreased abundance) differed significantly between the TGFβ2 and control groups (Supplementary Table S1) (Table 4).

### 3.4. Phosphorylated Proteins Are Enriched in the Mitophagy Pathway upon TGFβ2 Activation

GO and KEGG enrichment analyses are commonly used tools for gene functional annotation and biological interpretation [39]. To investigate the regulatory mechanism of protein phosphorylation in MEPM cells upon TGFβ2 activation, we performed functional enrichment analysis of DAPs. The GO functional annotation and GO enrichment analysis showed that there were 486 GO annotations among the 351 DAPPs between the TGFβ2 and control groups, including 350 involved in biological processes, 76 involved in molecular functions, and 60 involved in cellular components (Table 5, Supplementary Table S2, Supplementary Figure S1H). The GO functions were significantly enriched in processes such as the extracellular region, mitochondrial matrix, endoplasmic reticulum, and oxidation-reduction (Figure 4D).

The KEGG pathway analysis showed that 184 phosphoprotein pathways were significantly altered (Supplementary Table S3). The 8 most altered pathways were the mTOR, MAPK, ErbB, GnRH, HIF-1, Autophagy, and Mitophagy signaling pathways, but only the mTOR signaling pathway was statistically significant (Figure 4E). Based on the subcategories of KEGG, we performed hierarchical enrichment analysis on the pathways associated with phosphorylated proteins [40]. Pathway analysis revealed that the differentially phosphorylated proteins clustered most strongly within signal-transduction cascades, followed by transport and catabolism, and then translation (Supplementary Figure S1I).

We further performed GSEA analysis to evaluate the profile of the entire gene set to avoid missing potential biological signals due to threshold settings [41]. The enrichment score curve revealed that the hallmarks of TNFα signaling via NFκB, IL2-STAT5 signaling, and Heme metabolism were increased abundance, whereas the hallmarks of oxidative phosphorylation and EMT were decreased abundance in the TGFβ2-treated MEPM cells (Supplementary Figure S2A). The core genes on the right of the enrichment score curve were thought to contribute to this pathway or function.

Based on the pathway enrichment results, we further used a network diagram to visually display the gene-pathway changes. Our results showed that phosphorylated proteins upon TGFβ2 activation were mainly enriched in autophagy and its related pathways, and that autophagy was correlated with TGFβ, mTOR, P53, and other pathways. Within this network, Mki67, Dkc1, Mcm2, and Top2a emerged as central nodes, suggesting their potential regulatory importance (Supplementary Tables S4–S6, Supplementary Figure S2B). Prior research highlighted the involvement of Mki67 and Mcm2 in CP [42,43]. In contrast, the roles of Dkc1 and Top2a in palatal development remain unexplored, pointing to novel candidate genes for future investigation.

### 3.5. CSNK2A, MAPK, and CDK Are Key Kinases Under TGFβ2 Activation

MoMo (Modification Motifs), a tool in the MEME Suite, is used for post-translational modification site sequence analysis, which can identify and extract enriched amino acid sequence motifs from a large number of modified peptides, thereby inferring potential upstream kinases and regulatory mechanisms [44]. We further used the MoMo algorithm to analyze the data from the TGFβ2-treated group and the control group to identify their unique phosphorylation motifs. Our results showed that in the increased abundant phosphoproteins of the TGFβ2-treated group, a total of 7 serine (p-Ser) motifs and 1 threonine (p-Thr) motif were identified, including RxxS, SDxD, SDxE, SP, SxDE, SxEE, SxxxxD, and TP (Supplementary Figure S3A). These motifs were generally associated with the characteristics of acidic or acid-rich kinase substrates, suggesting that after TGFβ2 stimulation, kinases such as CDKs, MAPKs, and CaMKs might be activated, thereby driving processes such as cell cycle regulation, stress response, and autophagy.

**Figure 5:**
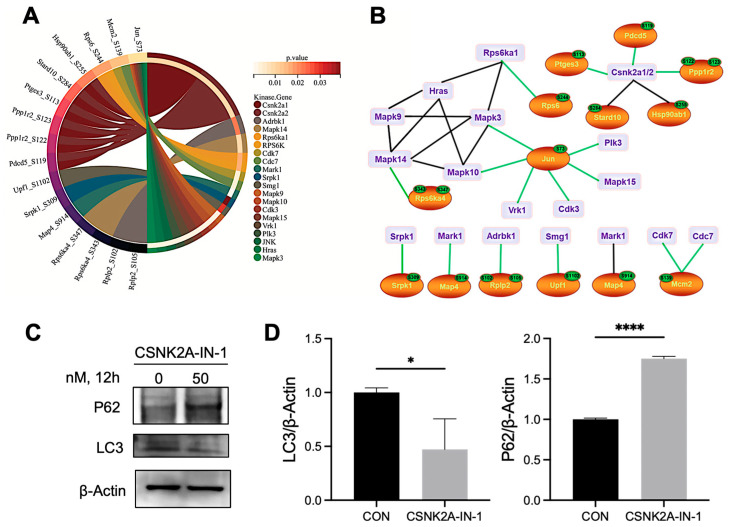
Identification of phosphorylation motifs and upstream kinases regulating TGFβ2-induced autophagy. (**A**) KSEA was performed to predict upstream kinases. The Circos plot illustrates the predicted relationships between activated kinases (outer segments) and their identified phosphoprotein substrates (inner links). (**B**) A curated kinase-substrate network focusing on the key kinases (e.g., CSNK2A) and their substrates related to autophagy and palatal development. (**C**,**D**) To validate the role of CSNK2A, MEPM cells were treated with a CSNK2A inhibitor (50 nM, 12 h). Representative Western blots (**C**) and corresponding quantification (**D**) show the protein levels of autophagy markers LC3 and P62. The Data are expressed from 3 biological replicates (**A**,**B**) or 3 biological replicates with 3 technical replicates each (**C**,**D**). Mean ± SD, * *p* < 0.05, **** *p* < 0.0001.

However, motif analysis alone cannot directly infer kinase activity. Therefore, we next performed KSEA to systematically evaluate kinase activity changes under TGFβ2 stimulation [45]. The KSEA identified 24 kinases that increased in abundance and 3 kinases that decreased in abundance in TGFβ2-treated MEPM cells (Supplementary Table S7). Notably, among all kinases with increased abundance, CSNK2A1/2 are the most activated substrates (Figure 5A). To further investigate the downstream effects of kinase activity under TGFβ2 stimulation, we systematically analyzed the phosphorylation of their substrates based on the KSEA results. CSNK2A induced the phosphorylation of Ptges3-S113, Stard10-S284, Hsp90ab1-S255, Ppp1r2-S122, Ppp1r2-S123, and Pdcd5-S119. Among these, previous studies have shown that Hsp90ab1-S255 phosphorylation is a key step in ATG1/ULK1-induced autophagy [46]. The association of other sites with autophagy requires further investigation. Moreover, the mitogen-activated protein kinases, including MAPK1, MAPK3, MAPK9, MAPK10, MAPK14, and MAPK15, and cyclin-dependent kinases, including CDK3 and CDK7, were activated in the TGFβ2-treated MEPM cells (Figure 5B).

Given that KSEA identified CSNK2A as a top activated kinase, we sought to confirm its role in autophagy using its specific inhibitor, CSNK2A-IN-1. As shown by the Western blot analysis, pharmacological inhibition of CSNK2A markedly suppressed autophagy (Figure 5C,D). Specifically, exposure to the inhibitor caused LC3-II to decline significantly, while p62 levels rose substantially. The accumulation of p62 indicates a blockage of autophagic degradation. These findings provide direct evidence that CSNK2A activity is required for TGFβ2-induced autophagy in MEPM cells, thereby validating the results from our phosphoproteomic and kinase activity analyses.

### 3.6. Kinase-Substrate Network Reveals Changes in MAPK and mTOR Pathways Under TGFβ2 Activation

**Figure 6:**
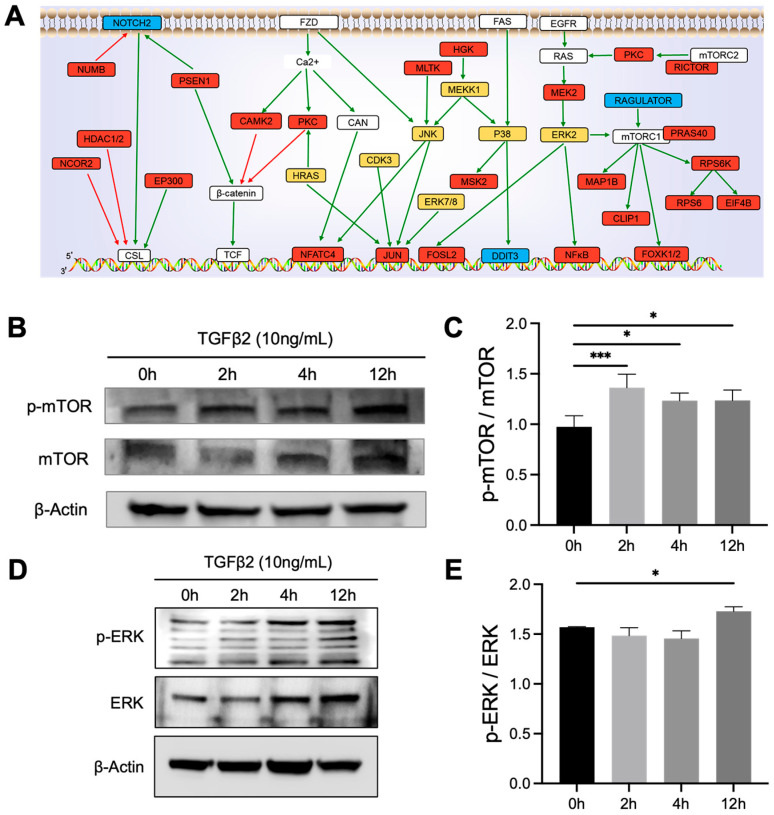
The biological network of TGFβ2-induced autophagy in MEPM cells constructed based on bioinformatics and KSEA. (**A**) The global protein–protein interaction network of differentially phosphorylated proteins identified in response to TGFβ2 treatment. (**B**–**E**) Experimental validation of key signaling pathway activation. E13.5 MEPM cells were treated with 10 ng/mL TGFβ2 for the indicated time points (0, 2, 4, 12 h). Representative Western blots and corresponding quantification show a significant increase in the phosphorylation of mTOR (**B**,**C**) and ERK (**D**,**E**) at the 12 h. The Data are expressed from 3 biological replicates (**A**) or 3 biological replicates with 3 technical replicates each (**B**–**E**). Mean ± SD, * *p* < 0.05, *** *p* < 0.001.

After using KSEA to analyze kinases and their substrates, we further integrated the relevant information to construct an autophagy-related signaling network under TGFβ2 activation. Our results showed that the activation of the autophagy process involves multiple signaling pathways, including the MAPK, mTOR, Wnt, and Notch pathways (Figure 6A, Supplementary Figure S3B).

In the Notch pathway, NOTCH2 mediated genes through the downstream transcription factor CSL, while also interacting with molecules such as PSEN1 and EP300. The Wnt pathway mediated Ca^2+^ signal transmission through FZD receptors, activating molecules such as CAMK2, PKC, and CAN, which then act on β-catenin and TCF, ultimately regulating the activity of transcription factors NFATC4 and JUN. The MAPK pathway mainly activated a multi-level kinase cascade (e.g., RAS-MEK-ERK, HGK-MLTK-MEKK1-JNK, P38, and ERK7/8) through FAS and EGFR receptors, and further acted on key transcription factors such as JUN, FOSL2, DDIT3, and NFκB to mediate the transcription of autophagy-related genes. The mTOR pathway integrated signals through the mTORC1 and mTORC2 complexes, with molecules such as RAGULATOR, RICTOR, PRAS40, RPS6K, and EIF4B involved, ultimately affecting cell metabolism and autophagy activity.

To confirm our network-based predictions, we conducted in vitro experiments. Western blot results demonstrated that the phosphorylation ratios of p-mTOR/mTOR and p-ERK/ERK were significantly elevated at 12 h following TGFβ2 administration (Figure 6B–E). This temporal activation profile provides direct evidence for the involvement of the mTOR and MAPK pathways in the TGFβ2-induced autophagic process, aligning precisely with the findings from our network analysis.

## 4. Discussion

Autophagy plays a key role in various physiological and pathological conditions and embryonic development [18,19]. Shi et al. further confirmed that embryonic autophagy maintains the stemness and osteogenic potential of mesenchymal stem cells in the palatal shelves of mouse embryos via the PTEN-Akt-mTOR signaling pathway, highlighting its key role in normal palatogenesis [47]. Although the signaling mechanism of TGFβ-induced autophagy has been described [48,49,50], the specific molecular events governing this process is partially ambiguous. Our findings confirmed that autophagy is a key event during E13.5–E15.5. Specifically, our IHC staining showed increased expression of the autophagic markers BECLIN-1 and LC3 as the palatal shelves fused. To further explore this process, we performed pathway enrichment analyses on the existing bulk-seq data. Bulk-seq results confirmed the enrichment of the autophagy pathway and additionally revealed a significant co-enrichment of the TGFβ signaling pathway within the same highly upregulated gene cluster. This prompted us to further investigate the specific mechanisms that how TGFβ controls autophagy.

During mammalian development, the secondary palate forms as the palatal shelves grow, elevate, and move toward each other, followed by adhesion of the medial edge epithelium (MEE) and elimination of the Midline Epithelial Seam (MES), which allows the shelves to fuse [51]. Disruption of any step in this sequence results in CP, a common craniofacial anomaly. Prior research demonstrated the pivotal role of TGFβ isoforms during palatogenesis [52]. While TGFβ1 and TGFβ3 are mainly found in MEE cells, mediating epithelial adhesion and MES disintegration, respectively [53,54,55], TGFβ2 is present in the palatal mesenchyme from E13.5 and remains detectable even after MES disappearance [56,57]. This distinct spatial and temporal pattern of TGFβ2 in the mesenchymal cells indicates a unique role in regulating their functions. Considering that protein phosphorylation is a key posttranslational modification governing cell signaling, we propose that TGFβ2 modulates the functions of palatal mesenchymal cells via specific phosphorylation events. To examine this, we set out to assess changes in protein phosphorylation in MEPM cells following TGFβ2 treatment.

Protein phosphorylation, a prevalent and vital posttranslational modification, plays a pivotal role in numerous cellular processes [58], including those essential for embryonic [59] and palatal development [60]. Given the known and significant phosphorylation changes that occur throughout embryonic development [61,62,63], we believe that a comprehensive understanding of the phosphorylation landscape is critical to fully elucidate the mechanisms behind TGFβ2-induced autophagy during palate development. Consequently, we employed TiO2-based phosphopeptides enrichment coupled with high-accuracy MS characterization to generate a detailed signaling network, which will help define the key phosphorylation events controlling this process.

Our analysis of differential protein phosphorylation indicated that the SMAD-independent signaling pathway were engaged in response to TGFβ2, promoting proper autophagy. This emphasis on SMAD-independent pathways is supported by our finding that we did not detect altered phosphorylation of SMADs in our TMT analysis. One plausible explanation for this is that the phosphorylation of SMAD molecules is a highly transient event, occurring in the initial stages of TGFβ signaling. Phosphorylated Smads rapidly translocate into the nucleus to regulate target genes [64] and are then rapidly degraded [65,66]. The ephemeral nature of this process likely explains why their phosphorylation was not captured in our analysis, highlighting the predominant role of SMAD-independent pathways in mediating TGFβ2-induced autophagy.

In addition, KEGG analysis further highlighted the mTOR pathway as a key regulatory pathway, with critical components including Prkaa1, Rps6, Eif4b, Pras40, Clip1, Rictor, Lamtor1, Map2k2, and Mapk1. Our Western blot analysis in vitro corroborated this observation, showing a significant increase in the p-mTOR after TGFβ2 treatment over 12h, confirming pathway activation. This finding aligns with previous study showing that activation of the mTOR pathway via the PTEN/AKT/mTOR axis maintains the stemness of MEPM cell [47]. However, because conventional pathway analyses like KEGG often overlook important changes in less significantly altered genes, we extended our investigation using GSEA to achieve a more comprehensive perspective. Notably, GSEA results revealed additional biological processes beyond the mTOR pathway. We observed a significant reduction in oxidative phosphorylation in MEPM cells after TGFβ2 treatment, implying decreased cellular energy metabolism efficiency. While oxidative phosphorylation is more energy-efficient than glycolysis, glycolysis provides safer biomass production [67]. The balance between these two pathways is critical for numerous biological processes, including palate development. Moreover, EMT levels were found to decline. Although TGFβ2 is a known inducer of EMT, our findings suggest that in this specific context, its pro-EMT effect may be restrained by certain regulatory mechanisms [68]. Furthermore, our attention was drawn to the up-regulation of heme metabolism, a process rarely discussed in the context of palatal development. Since intrauterine hemorrhage and heme exposure can initiate oxidative and inflammatory stress, leading to increased incidence of cleft palate [69], this finding points to a novel link between metabolic regulation and palatogenesis.

Beyond clarifying biological mechanisms, our study also underscored the potential of phosphoproteomics in identifying diagnostic markers. Phosphoproteomics is a powerful approach for detecting kinase activities and phosphorylation sites that serve as disease biomarkers. For example, FLT3 phosphorylation serves as a biomarker for acute myeloid leukemia [70], and tyrosine phosphorylation networks involving EGFR and ErbB3 predict drug resistance in colorectal cancer [71]. In the context of CP, where autophagy dysregulation is a known pathogenic factor [72,73], establishing molecular indicators is crucial. To this end, our KSEA analysis showed CSNK2A (CK2) is the most activated substrate, characterized by the significant enrichment of substrates containing distinct motifs such as -sDxE-, -SxEE-, and -SDxD-. Previous study showed that the inhibition of CK2 disrupts the ATG12-ATG5-ATG16L1 complex and suppresses autophagy [74]. We also found inhibition of CSNK2A markedly suppressed TGFβ2-induced autophagy. In addition, CSNK2A-targeting agents such as CX-4945 exhibit favorable bioavailability and are currently under clinical evaluation for hematologic and solid malignancies [75]. Together, these observations suggest that CSNK2A may serve as a molecular indicator of dysregulated developmental signaling pathways.

In addition, our analysis highlighted G protein-coupled receptor kinase (GRK2, encoded by the Adrbk1 gene), which presented the highest Z-score. GRK2 is a serine/threonine protein kinase that regulates beta-adrenergic signaling and recognize substrates with an -SP- motif [76]. Furthermore, we identified RPS6K, a serine/threonine kinase in the Ras-activated signaling cascade, whose phosphotransferase activity is directed towards substrates containing an -RxxS- motif at the phosphorylation site [77]. These findings suggest that multiple kinase cascades, particularly those independent of the classical SMAD pathway, are mobilized by TGFβ2 to coordinate the complex phosphorylation events necessary for proper cellular responses during palatogenesis.

Beyond kinases, TGFβ2-induced autophagy was accompanied by notable changes in the key transcription factors, including JUN, FOSL2, NFATC4, DDIT3, NFκB, FOXK1, and FOXK2. These molecules likely serve as downstream effectors that translate the phosphorylation signals into specific gene patterns. Notably, NFATC4, DDIT3, FOXK1, and FOXK2 have not been previously reported in studies related to CP, highlighting their potential as novel regulatory players and warrants further investigation.

Our findings provide new insights into the functional mechanisms of these transcription factors in palatogenesis. For instance, NFATC4 is tightly enriched by the calcium/calmodulin-dependent phosphatase calcineurin and is known to bind cooperatively with AP-1 family transcription factors (Fos/Jun) [78]. The differential expression of JUN and FOSL2 in our analysis supports a potential NFATC4: AP-1 complex-mediated regulation. Additionally, we found DNA damage-inducible transcript 3 (DDIT3), a typical ER stress marker that is essential for the transcriptional induction of autophagy genes like ATG10 and ATG5 in response to stress [79,80]. This suggested a link between TGFβ2, ER stress, and autophagy in palatal cells. Finally, Forkhead transcription factors FoxK1 and FoxK2, which are downstream targets of the Akt-mTOR pathway, are known to control apoptosis, metabolism, and mitochondrial function [81]. The activation of the Akt-mTOR pathway and the FoxK1/K2 further strengthens the connection between this signaling axis and the downstream transcriptional events in MEPM cells.

TMT-based phosphoproteomic analysis has inherent limitations. Because TMT labeling and phosphopeptide enrichment involve processing under non-cold conditions, a certain degree of residual loss of phosphorylation cannot be completely avoided, even though protein lysis was performed under strongly denaturing conditions with the immediate addition of broad-spectrum phosphatase inhibitors and all samples were processed in parallel using an identical and strictly controlled workflow [82]. However, this effect occurs systematically across all experimental groups. As a result, its impact on relative quantitative comparisons between groups is limited and unlikely to affect the reliability of the core conclusions of this study. Furthermore, our current dataset does not capture combinatorial modifications associated with oxidation, acetylation, or ubiquitination. Future investigations employing top-down proteomics or integrated multi-PTM strategies will be essential to resolve these specific proteoforms and achieve a more comprehensive understanding of proteome complexity. Furthermore, the majority of our mechanistic investigations were conducted in vitro using MEPM cells. While this model is effective for studying specific cellular responses, it may not fully replicate the intricate spatiotemporal regulation that occurs within the developing palate in vivo.

Despite the limitation, our study provides a detailed map of the phosphorylation landscape downstream of exogenous TGFβ2, revealing its crucial role in activating autophagy during palatal formation. We found that TGFβ2 mediated the key phosphorylated proteins, particularly through SMAD-independent signaling pathways. This mechanistic insight into how protein phosphorylation controls autophagy is essential for understanding the molecular basis of normal palatogenesis. Therefore, our findings offer valuable targets for future therapeutic strategies aimed at preventing or alleviating congenital CP.

## Figures and Tables

**Table 1:** Antibodies used for Western blot.

Antibodies	Source	Identifier	Dilution Ratio
LC3	Cell Signaling Technology	2775	1:5000
P62	Abcam	ab109012	1:2000
Beclin-1	Cell Signaling Technology	3494	1:2000
p-ERK	Abclonal	AP0974	1:1000
ERK	Proteintech	66192-1-Ig	1:10,000
p-mTOR	Cell Signaling Technology	2971	1:1000
mTOR	Proteintech	66888-1-Ig	1:5000
β-Actin	Proteintech	66009-1-Ig	1:5000
HRP-conjugated goat anti-mouse secondary antibodies	Proteintech	SA00001-1	1:5000
HRP-conjugated goat anti-rabbit secondary antibodies	Abclonal	AS014	1:5000

**Table 2:** The parameters and descriptions for database identification and quantitative analysis.

Type	Identification
Protein Database	Uniprot_MusMusculus_17027_20210210
Enzyme	Trypsin
Max Missed Cleavages	2
Instrument	ESI-Orbitrap
Precursor Mass Tolerance	±10 ppm
Fragment Mass Tolerance	0.05 Da
Use Average Precursor Mass	False
Modification Groups from Quan Method	TMT 16plex
Dynamic modifications	Oxidation (M); Acetyl (Protein N-term); Phosphorylation (S, T, Y)
Static modifications	Carbamidomethyl (C)
Database pattern for calculating FDR	decoy
Peptide FDR	≤0.01

**Table 3:** The numbers of the identification and quantification of phosphorylated protein.

Type	Identification	Phosphorylation
Peptides	23,471	6339
Proteins	3952	2195

**Table 4:** Statistics of differentially abundant phosphorylated peptides and protein.

Type	Increased Abundance (>1.2)	Decreased Abundance (<0.83)	Total
Peptides	477	53	530
Proteins	315	37	351

**Table 5:** Statistics of functional annotation by GO analysis.

Type	Increased Abundance	Decreased Abundance	Total
Biological Process	165	185	350
Cellular Component	36	24	60
Molecular Function	42	34	76

## Data Availability

All datasets generated for this study are included in the article/Supplementary Materials; further inquiries can be directed to the corresponding author.

## Supplementary Materials

The following supporting information can be downloaded at: https://www.mdpi.com/article/10.3390/proteomes14010005/s1, Figure S1: Experimental workflow and characteristics of the quantitative phosphoproteomic analysis; Figure S2: Identification of autophagy-related signaling networks using GSEA; Figure S3: Phosphorylation motif analysis of the identified phosphopeptides. Table S1: Top upregulated phosphoproteins and their fold changes identified by phosphoproteomics; Table S2: GO enrichment analysis of differentially expressed phosphoproteins; Table S3: KEGG pathway enrichment analysis of differentially expressed phosphoproteins; Table S4: List of PPI and Evidence Scores Retrieved from STRING Database (Mus musculus); Table S5: Core modules and scores of the PPI network; Table S6: Identification of key hub genes using different centrality methods; Table S7: Top 10 hub genes ranked by eight topological analysis methods.

## Abbreviations

The following abbreviations are used in this manuscript.

CP	Cleft palate
TGFβ	Transforming growth factor-beta
Hh	Hedgehog
E	Embryonic day
LAMP2	Lysosome-associated membrane protein 2
LC3	Microtubule-associated protein 1 light chain 3 alpha
CQ	Chloroquine
MEPM	Mouse embryonic palatal mesenchymal
EMT	Epithelial-to-mesenchymal transition
H&E	Hematoxylin and Eosin
IHC	Immunohistochemistry
Bulk RNA-seq	Bulk RNA sequencing
DEGs	Differentially expressed genes
KEGG	Kyoto Encyclopedia of Genes and Genomes
TMT	Tandem mass tag
MS	Mass spectrometry
PCA	Principal component analysis
GO	Gene Ontology
GSEA	Gene set enrichment analysis
MoMo	Modification Motifs
KSEA	Kinase-substrate enrichment analysis
CK2	Casein Kinase 2
MAPK	Mitogen-activated protein kinase
CDK	Cyclin-dependent kinase
MEE	Medial edge epithelium
MES	Midline epithelial seam
DDIT3	DNA damage-inducible transcript 3
LC-MS/MS	Liquid chromatography-tandem mass spectrometry
PPI	Protein–protein interaction
MCODE	Molecular Complex Detection
