# Supplementary material for: Integrated Phosphoproteomics Identifies TGFβ-Dependent Phosphorylation Events Linking Kinase Signaling to Autophagy in Palatogenesis
Source: Proteomes. 2026 Jan 23;14(1):5. doi: 10.3390/proteomes14010005 (PMC12921946; doi:10.3390/proteomes14010005)
Supplement: Supplementary file 1 [file proteomes-14-00005-s001.zip › Source data for Western blots.pdf]

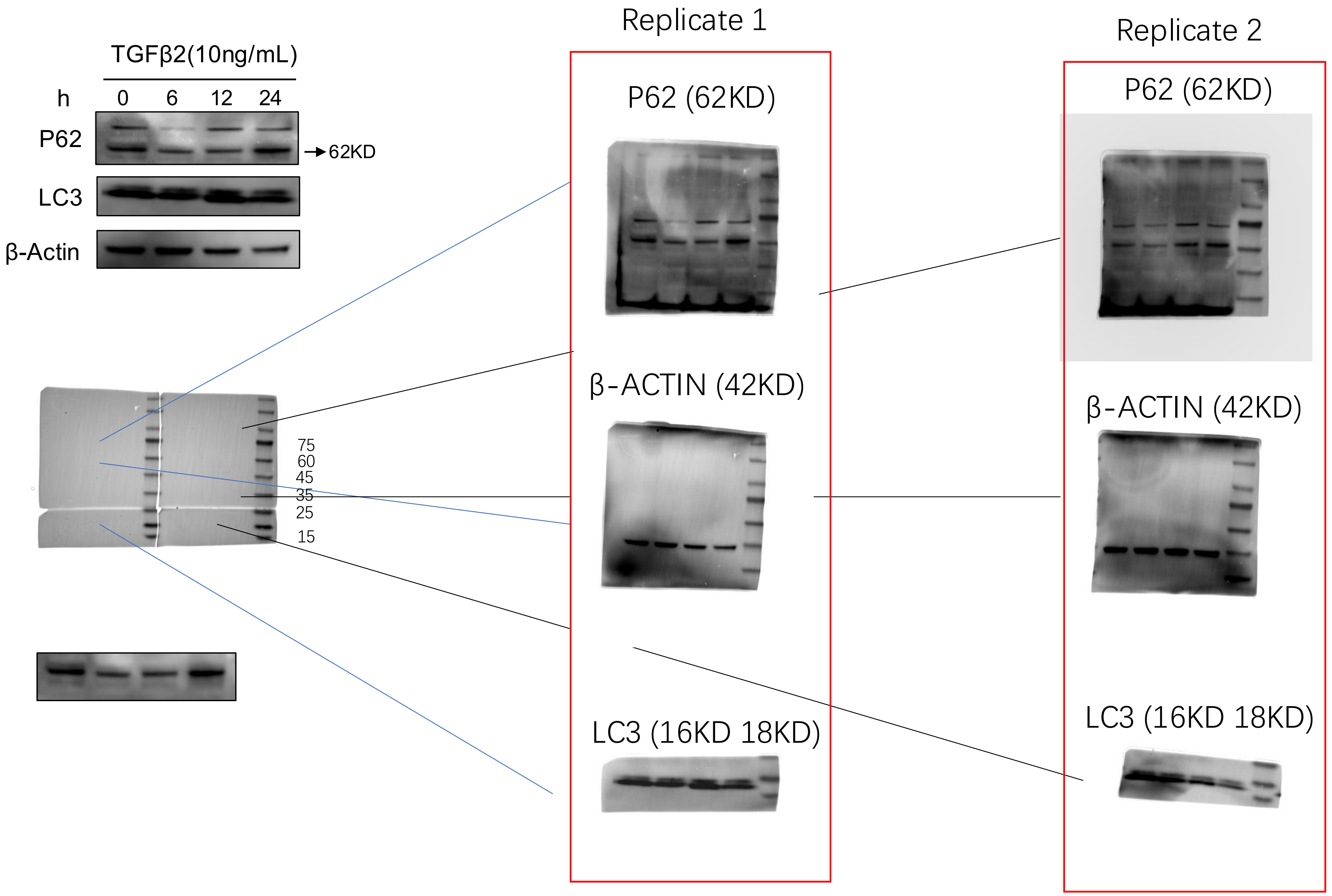

TGFβ2(10ng/mL)
h
0
6
12
24
P62
→62KD
LC3
β-Actin
75
60
45
35
25
15
Replicate 1
P62 (62KD)
β-ACTIN (42KD)
LC3 (16KD 18KD)
Replicate 2
P62 (62KD)
β-ACTIN (42KD)
LC3 (16KD 18KD)

Replicate 3

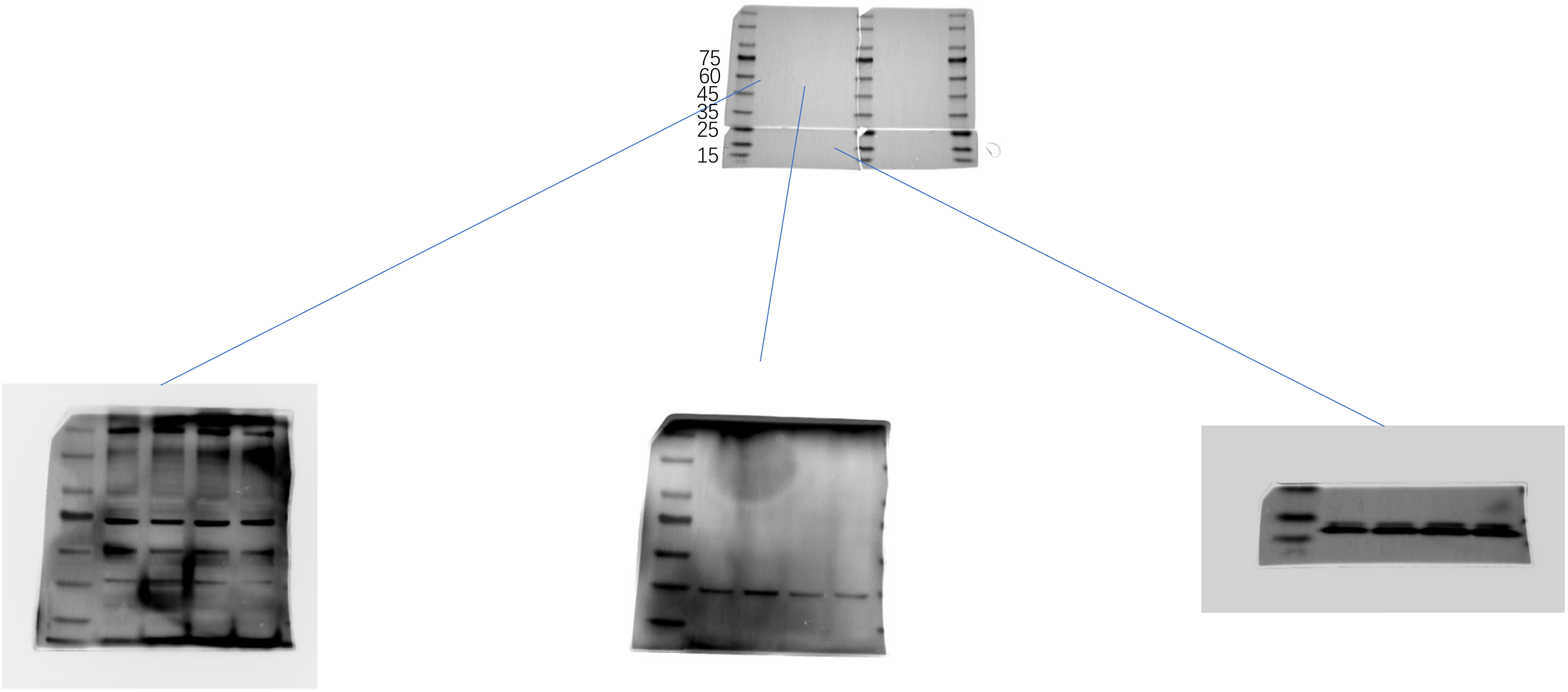

P62 (62KD)

β-ACTIN (42KD)

LC3 (16KD 18KD)

FIG 5C

Replicate 1

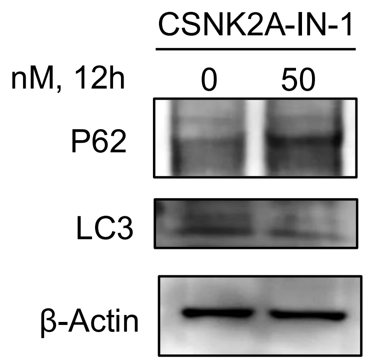

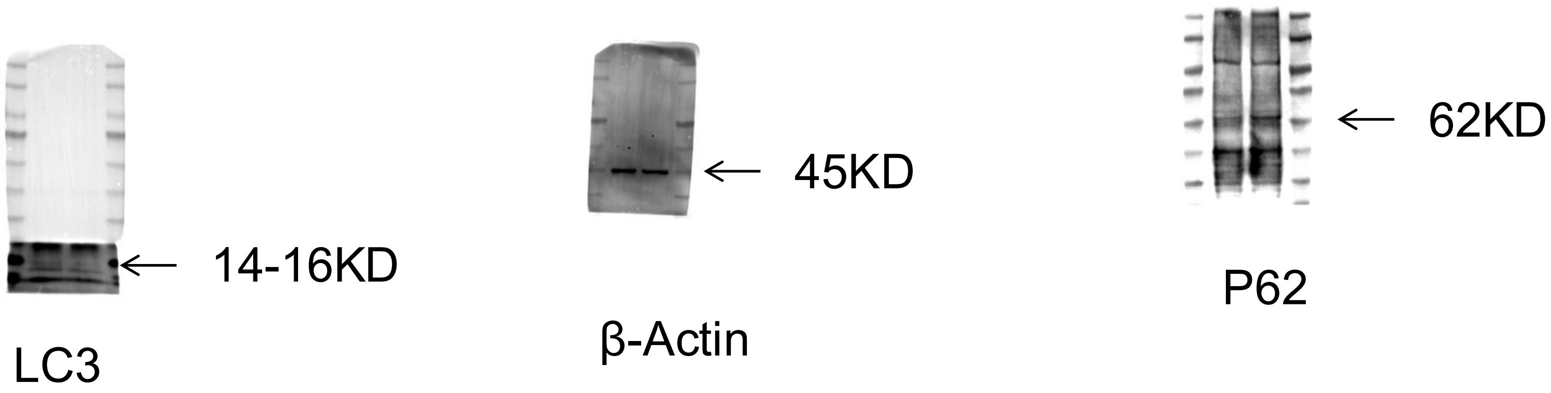

Replicate 2

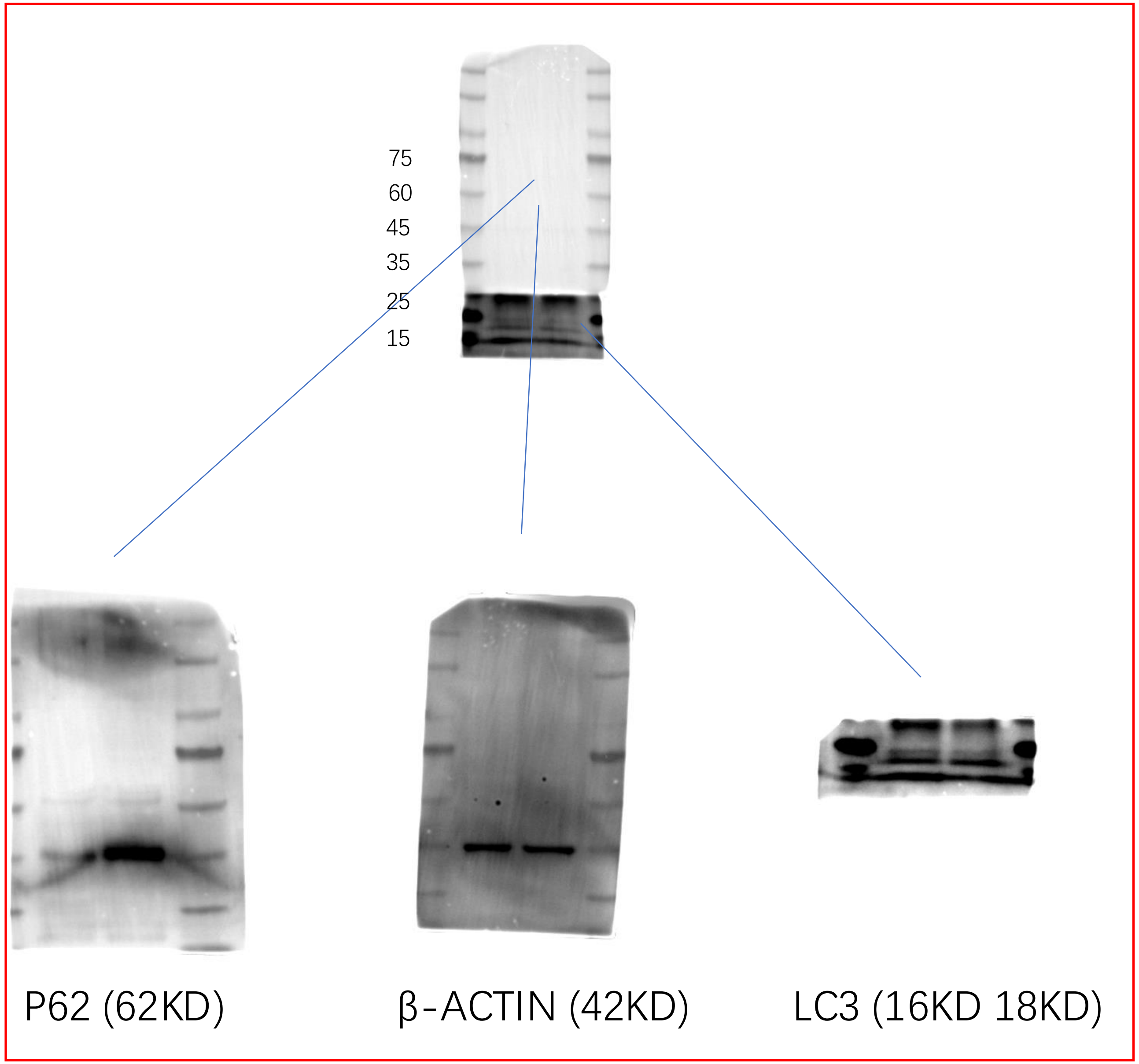

Replicate 1

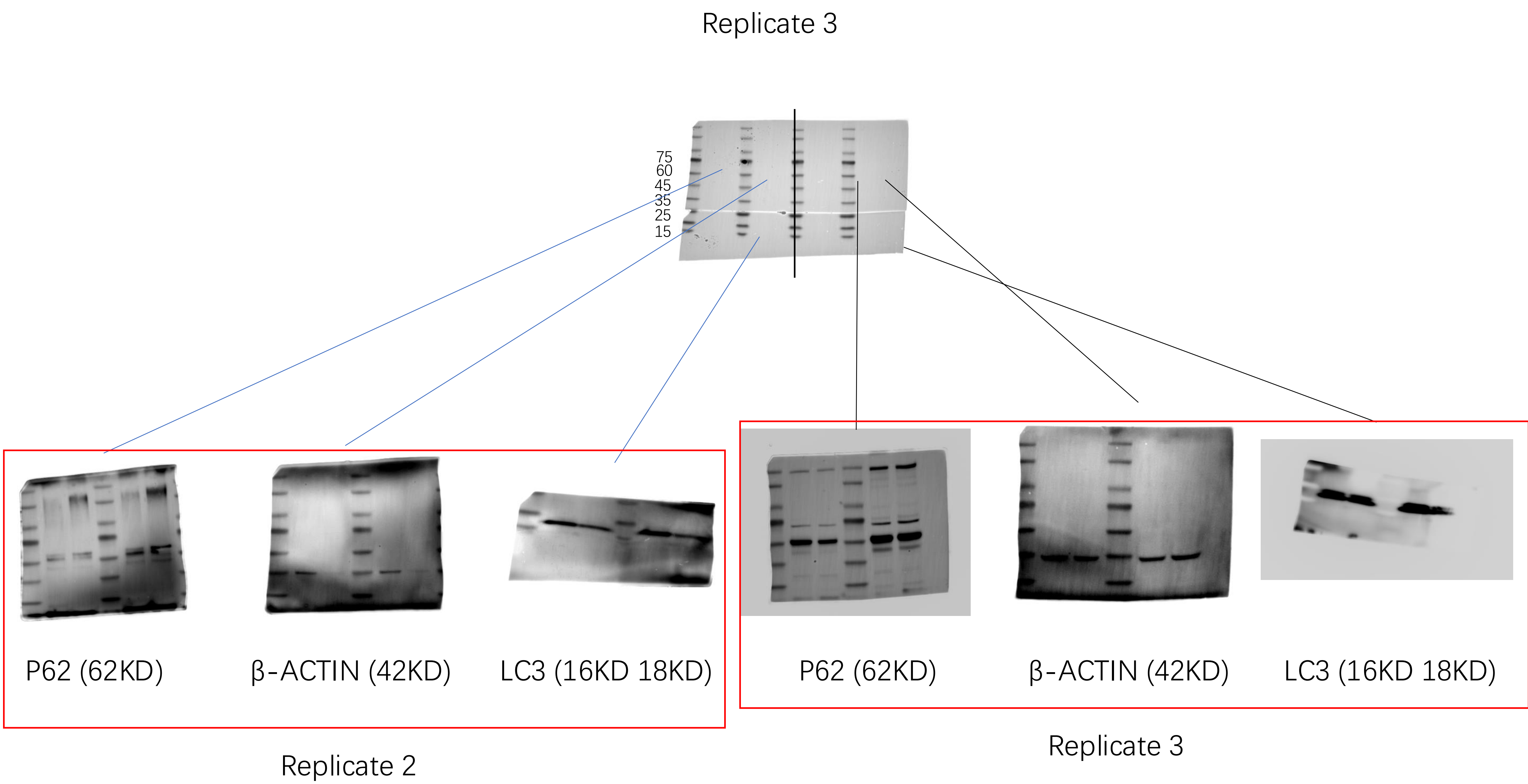
Replicate 3
75
60
45
35
25
15
P62 (62KD)
β-ACTIN (42KD)
LC3 (16KD 18KD)
Replicate 2
P62 (62KD)
β-ACTIN (42KD)
LC3 (16KD 18KD)
Replicate 3

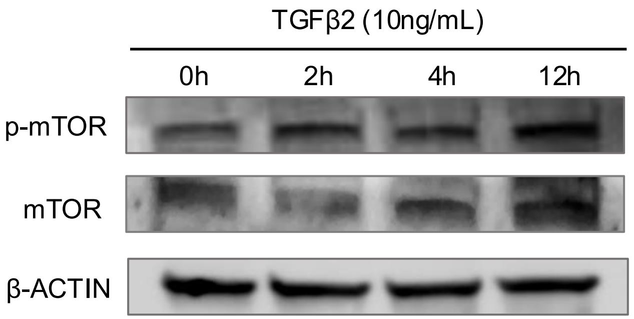

p-mTOR (289kd)

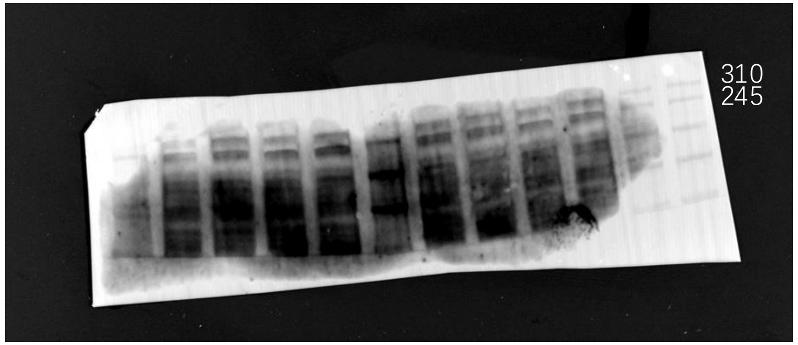

mTOR (289kd)

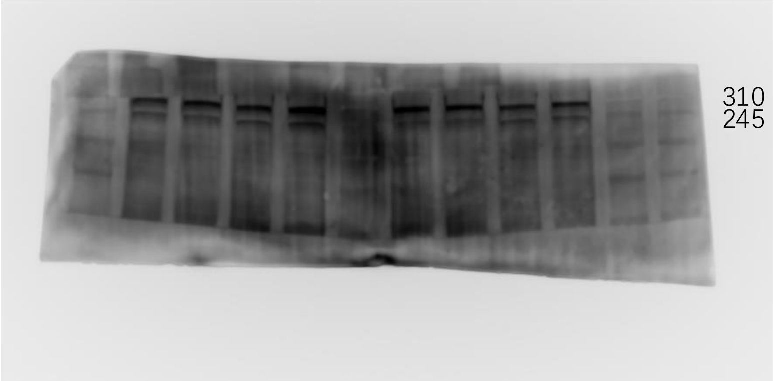

β-ACTIN (42kd)

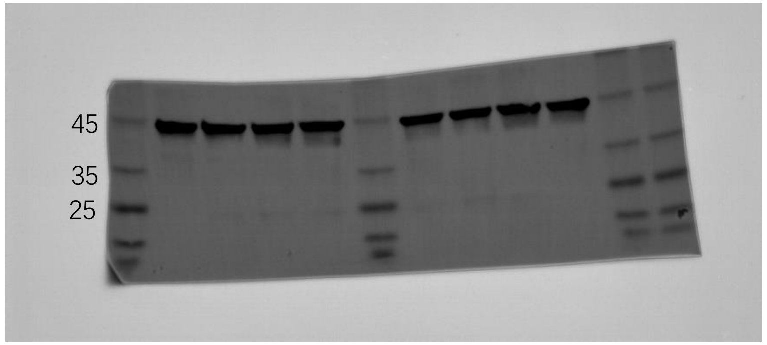

Replicate 1/2

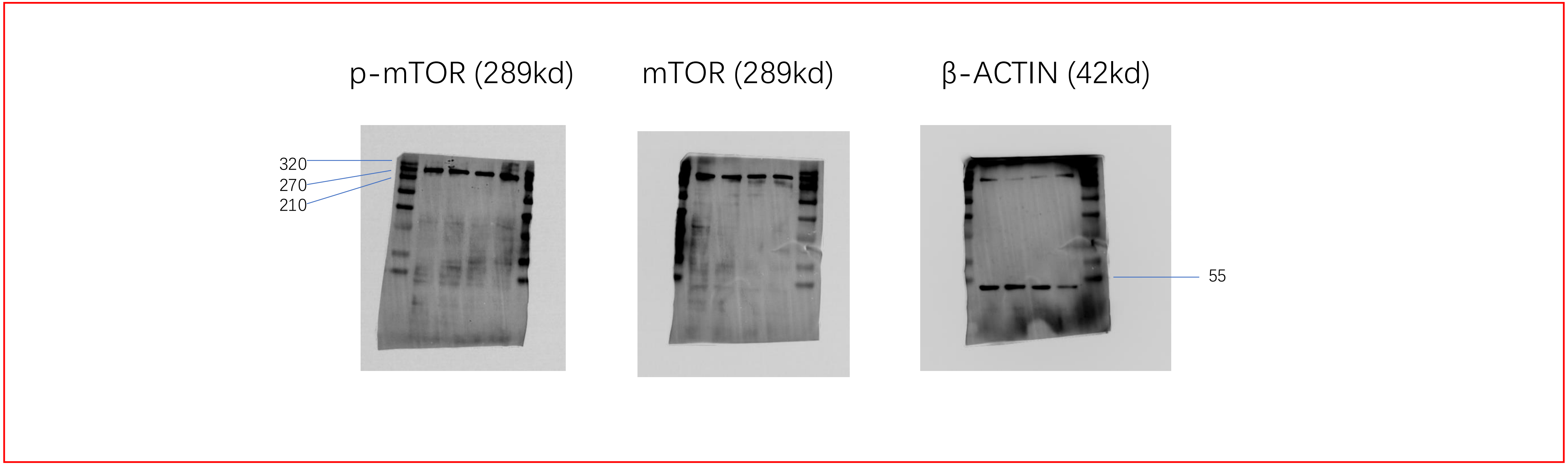

Replicate 3

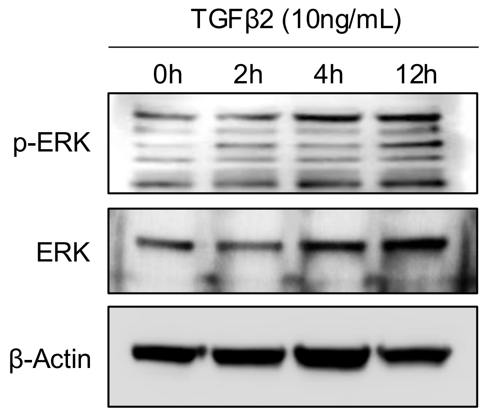

p-ERK (43KD)

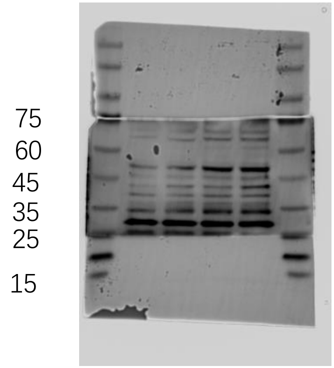

ERK (43KD)

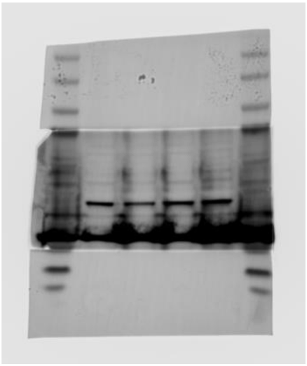

β-ACTIN (42KD)

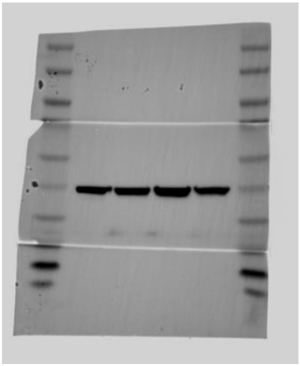

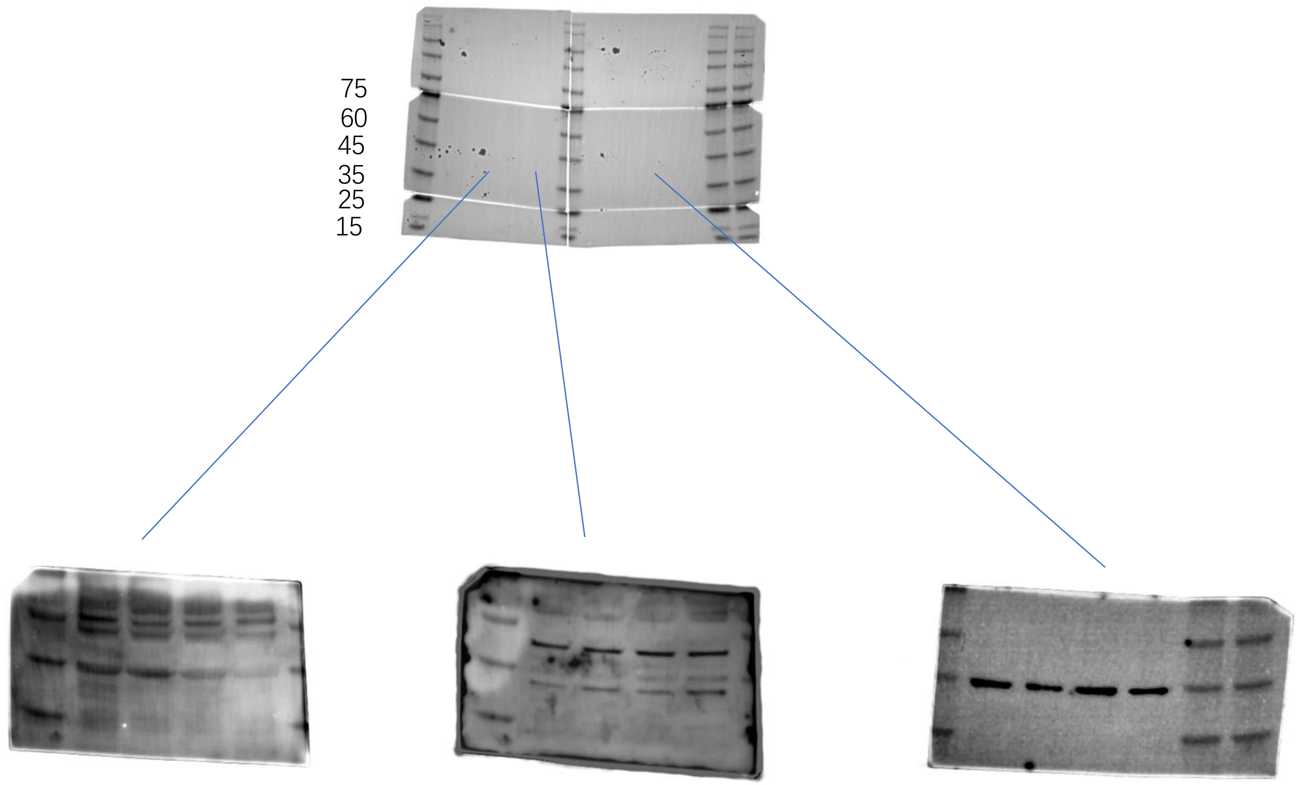

p-ERK (43KD) ERK (43KD) β-ACTIN (42KD)
