# Supplementary material for: Integrated Phosphoproteomics Identifies TGFβ-Dependent Phosphorylation Events Linking Kinase Signaling to Autophagy in Palatogenesis
Source: Proteomes. 2026 Jan 23;14(1):5. doi: 10.3390/proteomes14010005 (PMC12921946; doi:10.3390/proteomes14010005)
Supplement: Supplementary file 1 [file proteomes-14-00005-s001.zip › Supplementary Files/Supplementary Figures.pdf]

Supplementary Figure 1

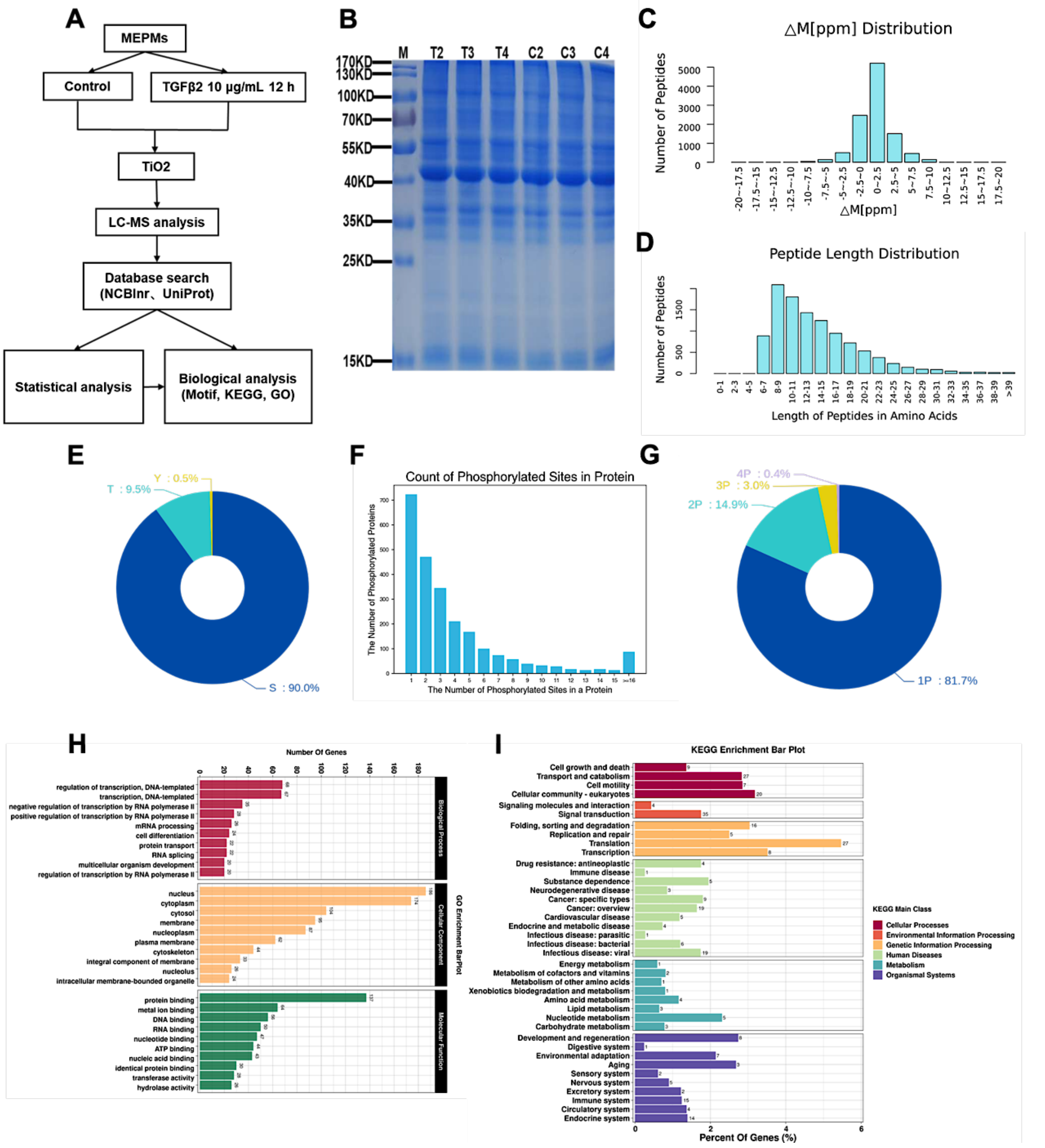

(A) Experimental scheme of the quantitative phosphoproteomic analysis; (B) The protein expression levels derived from the whole cell lysates of the Control group (C2-C4) and the TGFβ2 group (T2-T4) were determined by sodium dodecyl sulfate-polyacrylamide gel electrophoresis (SDS-PAGE) and Coomassie Brilliant Blue staining. (C) Mass deviation distribution of peptide segment; (D) Peptide sequence length distribution diagram. (E) The proportion of phosphoserine, phosphothreonine and phosphotyrosine. The pie charts depict the phosphopeptide and phosphosites distributions. (F-G) Number distribution of phosphopeptide modification sites. (H) GO enrichment analysis scatterplot. The x-axis shows the enrichment factors representing the significance and reliability of the phosphoproteins. (I) KEGG enrichment analysis barplot. The phosphorylated proteins were divided into 6 KEGG main

classes. The numbers adjacent to each bar are the number of differentially expressed phosphoproteins enriched in the term.

Supplementary Figure 2

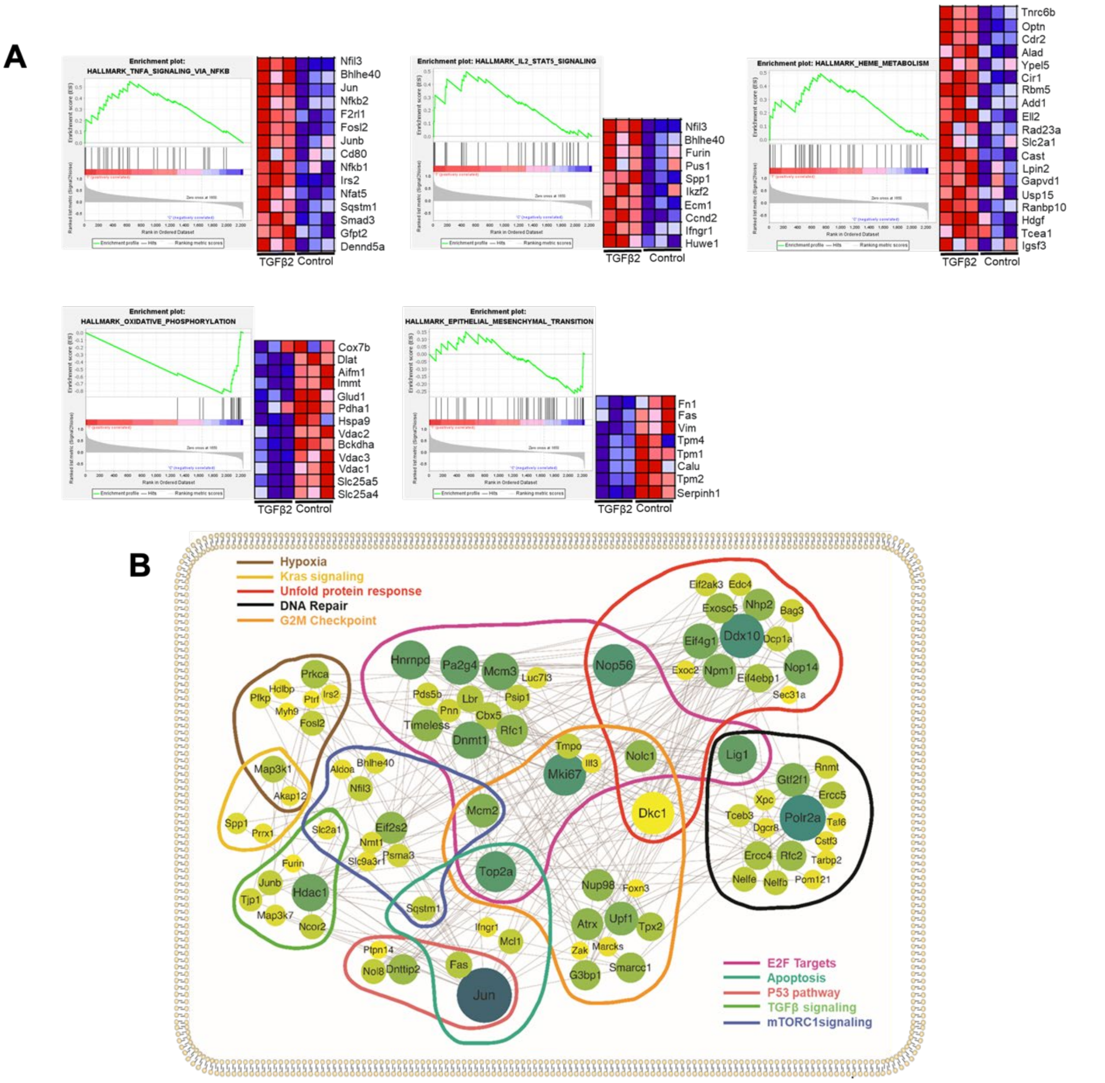

(A) GSEA displaying the most significantly enriched pathways in the TGFβ2-treated group. (B) Network visualization of autophagy-associated cellular processes identified from GSEA results. Nodes represent enriched gene sets (processes), and edges indicate the degree of gene overlap between them.

Supplementary Figure 3

A

Motif logo	Motif	Motif score	Fold increase	Foreground		Background		Regulation	E-value
				Matches	Size	Matches	Size		
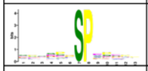	……SP…...	45.29	5.12	104	280	55984	55984	Up	2.5e-043
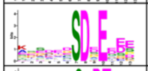	……SD.E…	44.90	24.06	22	176	3720	716020	Up	1.0e-020
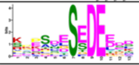	……S.DE…	28.69	25.79	14	154	2511	712300	Up	5.2e-013
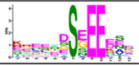	……S.EE…	22.21	16.48	18	140	5505	709789	Up	6.1e-014
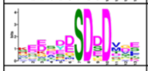	……SD.D…	22.90	31.55	15	122	2745	704284	Up	2.1e-015
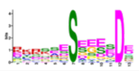	……S….D.	8.09	4.25	22	107	33955	701539	Up	3.8e-006
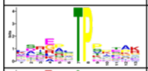	…….TP…..	13.52	8.93	18	30	32865	489379	Up	1.4e-011
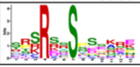	…R..S……	8.46	5.94	16	46	45176	772004	Down	1.6e-006

B

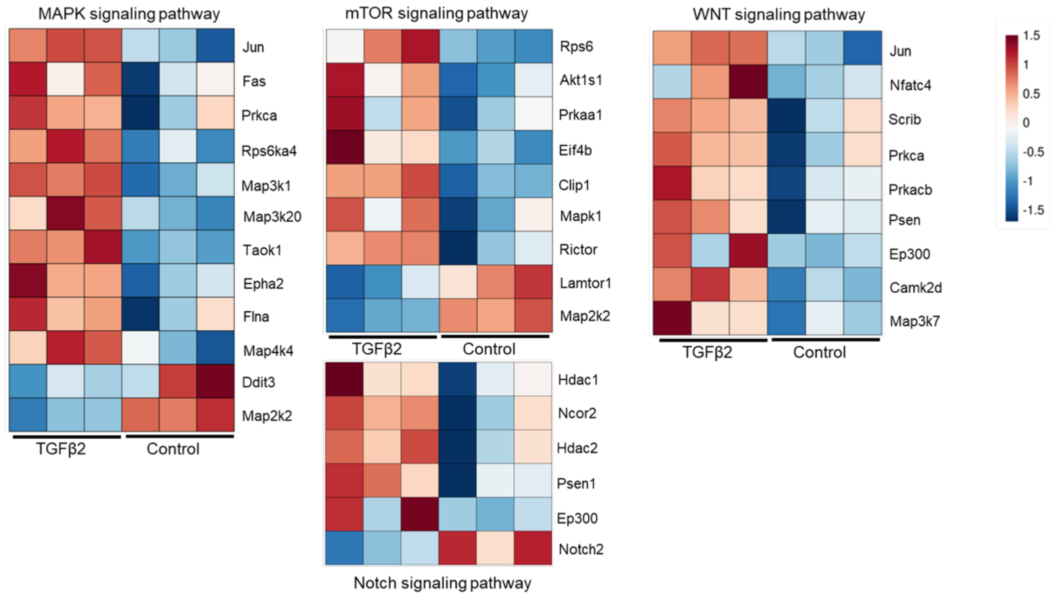

(A) Phosphorylation motif analysis of the identified phosphopeptides. The logos show the most overrepresented sequence motifs surrounding the phosphorylated serine (S)/threonine (T) residues. The tables show statistics for foreground (phosphorylated peptides) versus background (all identified peptides). Logos were generated using the MoMo tool.
